# CT-guided transthoracic core needle biopsies of focal pleural lesions smaller than 10 mm: a retrospective study

**DOI:** 10.1186/s40644-023-00569-4

**Published:** 2023-05-22

**Authors:** Melita Kukuljan, Ena Mršić, Eduard Oštarijaš

**Affiliations:** 1 Department of Radiology, Rijeka Clinical Hospital Centre, University of Rijeka, Rijeka, Croatia; 2 Doctoral School of Pharmacological and Pharmaceutical Sciences, University of Pécs Medical School, Pécs, Hungary

**Keywords:** CT, Transthoracic core needle biopsy, Pleura, Pleural lesions

## Abstract

**Background:**

CT-guided transthoracic core needle biopsy (TCNB) is a minimally invasive diagnostic procedure and a useful radiological method for diagnosing pleural lesions smaller than 10 mm in the presence of loculated pleural effusion. The purpose of this study was to retrospectively assess the diagnostic accuracy of CT-guided TCNB of small pleural lesions and determine the incidence of complications.

**Methods:**

This retrospective study included a total of 56 patients (45 men and 11 women; mean [± SD] age, 71.84 ± 10.11 years) with small costal pleural lesions (thickness of < 10 mm) who underwent TCNB performed at the Department of Radiology from January 2015 to July 2021. One of the inclusion criteria for this study was a loculated pleural effusion greater than 20 mm, with a nondiagnostic cytological analysis. Sensitivity, specificity and positive as well as negative predictive values (PPV, NPV) were calculated.

**Results:**

The sensitivity of CT-guided TCNB for the diagnosis of small pleural lesions in this study was 84.6% (33 of 39), specificity 100% (17 of 17), PPV 100% (33 of 33), and NPV 73.9% (17 of 23), while diagnostic accuracy was 89.3% (50 of 56). The overall diagnostic contribution of TCNB in our study is comparable with the results of other recent reports. Loculated pleural effusion was considered a protective factor since no complications were noted.

**Conclusion:**

CT-guided transthoracic core needle biopsy (TCNB) is an accurate diagnostic method for small suspected pleural lesions with a near-zero complication rate in the presence of loculated pleural effusion.

## Background

Pleural diseases are common, affecting more than 0.3% of the population yearly. They originate from a wide range of pathologies; therefore, a systematic approach to diagnosis and treatment is required [1]. Most common solid pleural lesions can be divided into benign (such as fibrous pleural thickening, solitary fibrous tumour, and lipoma) and malignant plaques (such as metastases, malignant mesothelioma, lymphoma, and Askin tumour) [2, 3]. The vast majority of pleural neoplasms invade the pleura secondarily, while primary pleural neoplasms are less common [4]. Malignant pleural mesothelioma is an aggressive primary pleural tumour and is most associated with previous asbestos exposure [5]. Treatment and prognosis vary considerably, so a definite histopathological diagnosis is required for planning a treatment strategy.

Pleural effusion is the most common, clinically important secondary finding caused by pleural lesions. Histologically, effusion can be divided into transudate and exudate. The latter is frequently caused by infection, infarction, or malignancy [3]. Usually, a pleural cytologic examination is an initial step in the diagnostic work-up.

The diagnostic yield of cytologic analysis of thoracocentesis-obtained pleural effusion is 40–60%, while the sensitivity of pleural fluid cytology is 60%, which is relatively low [6]. Also, there is a considerable variation in the sensitivity of cytological assessment of pleural effusion, with significantly lower sensitivity for mesothelioma and haematological malignancies than for adenocarcinoma [7–9]. The diagnostic yield of the above mentioned examination can be improved by repeated thoracocentesis, especially when combined with a percutaneous pleural biopsy [10].

An image-guided transthoracic biopsy is a widely used, safe, and accurate diagnostic procedure for the diagnosis of pleural lesions. It can be performed under the guidance of CT or ultrasound (US). The advantages of US are real-time multiplanar monitoring and the absence of ionising radiation. US-guided transthoracic biopsy of pleural lesions is an effective radiological diagnostic method with excellent diagnostic accuracy for pleural lesions smaller than 20 mm, ranging from 66.7 to 97.1% [11]. Others [12] have suggested that US guidance should be considered for biopsy of peripheral lung and pleural lesions larger than 10 mm.

CT is a guidance modality of choice for transthoracic biopsy of small pleural lesions as a minimally invasive diagnostic procedure with high diagnostic contribution and an acceptably low complication rate [13]. The purpose of this study was to assess the previously mentioned statements for CT-guided TCNB of small pleural lesions with the presence of loculated pleural effusion as the protective factor for complications.

## Methods

The ethics committee of the Rijeka University Clinical Hospital Centre approved the conduct of this retrospective study. Written informed consent from patients was not required since this study was considered a review of clinical practice.

### Study cohort

This retrospective study included 56 patients who underwent CT-guided transthoracic pleural biopsy at the Department of Radiology, Rijeka University Clinical Hospital Centre, from January 2015 to July 2021. Out of a total number of patients, 80.4% (45 of 56) were male (age 72.93 ± 8.63 years), and 19.6% (11 of 56) were female (age 67.36 ± 14.39 years). The mean patient age was 71.84 ± 10.11 years (range 43–86).

During the period of 6 years, a total of 158 CT-guided transthoracic biopsies of pleural lesions were performed at our institution. The total number of patients excluded from the study was 102 for the following reasons: pleural lesions with thickness measuring 10 mm or more (n = 58), patients who do not have loculated pleural effusion greater than 20 mm (n = 33), and less than 12 months of follow-up after the procedure (n = 11).

Inclusive criteria for this study were small lesions (lamellar or spindle-shaped plaque) of costal pleura with thickness up to 10 mm, and the presence of loculated, gravity independent, pleural effusion, greater than 20 mm, localised immediately adjacent to the target lesion. Due to pleural effusion, the needle did not penetrate the visceral sheet of the pleura nor did it enter the lung parenchyma, thus avoiding the most common complications such as pneumothorax and needle tract bleeding. An additional inclusive criterion was a non-diagnostic cytological analysis of pleural effusion.

We collected patient data (age and sex) consecutively from the hospital information system (Ibis), while TCNB data (lesion size, needle size, number of tissue sampling) were collected from the picture archiving and communication system (PACS).

### Biopsy procedure

The multidisciplinary team (radiologist, pulmonologist, thoracic surgeon, pathologist, and oncologist) indicated this diagnostic procedure for suspected pleural malignancy. The interventional radiologist estimated if the pleural lesion was suitable for the biopsy based on a previously performed diagnostic contrast-enhanced chest CT. On the day of the intervention, every patient was admitted to day rehabilitative and curative care at the Department of Pulmonology.

Criteria for the biopsy procedure included the patient’s ability to cooperate adequately and tolerate the supine position. Also, a valid coagulation test (prothrombin time (PT), international normalised ratio (INR), and partial thromboplastin time (PTT)) was required as well as a signed informed consent.

All interventions included in this study were performed by two interventional thoracic radiologists, one (KM) with 20 years of experience and the other with three years of experience in TCNB.

Patient’s position (pronation, supination, or lateral decubitus) was determined based on the lesion localisation.

The procedure begins with a CT scanogram on which a scanning field was determined that encompasses only that part of the thorax in which the target lesion was located. Due to radiation protection, the narrowest possible field was chosen. All biopsies were performed without contrast agents, under the guidance of CT Siemens Somatom Definition AS (128), with a layer thickness of 2 mm.

The puncture site was labelled with a radiopaque marker, followed by preprocedural cleansing of the biopsy site with an antiseptic agent and a subsequent subcutaneous injection of local anaesthetic (2 mL of 2% lidocaine).

**Fig. 1:**
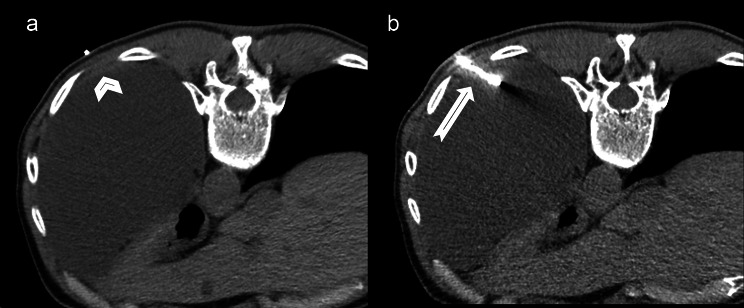
Axial scan of a chest CT obtained during CT-guided transthoracic biopsy (histopathological diagnosis: epithelioid mesothelioma) **1a**: radiopaque marker immediately next to a 5 mm thick solid pleural lesion (arrowhead) **1b**: biopsy needle (arrow)

**Fig. 2:**
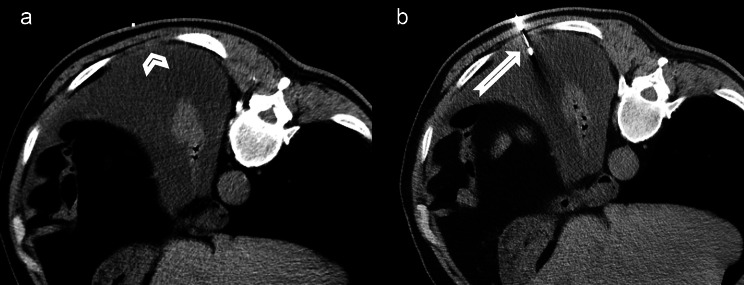
Axial scan of a chest CT obtained during CT-guided transthoracic biopsy (histopathological diagnosis: epithelioid mesothelioma) **2a**: radiopaque marker immediately next to a 4 mm thick solid pleural lesion (arrowhead) **2b**: biopsy needle (arrow)

After the skin incision, the pleural biopsy was performed with a single needle technique, 16-gauge semi-automated non-coaxial core biopsy needle (Original TEMNO™ Biopsy Device). The lesion was accessed along the upper margin of the lower rib to avoid damage to the vascular and nerve structures. A biopsy needle was led step-by-step to the pleural lesion; after each step, a CT scan was performed to check the needle position. Each needle manipulation and CT scan were performed only during suspended respiration. The number of punctures depended on the specimen quality, resulting in one or two samples. Since at our institution it is not possible to organise the presence of a cytopathologist during the biopsy, we evaluated the quality of the sample based on the morphology, whether the sample was disintegrated, and whether it dissolved in formalin. Two biopsy passes were performed in 47 (83.9%) of patients, while one pleural puncture was performed in only 9 (16.1%) of patients. The obtained tissue cylinders were sent for pathological analysis. After the procedure, a control non-contrast CT scan was performed to detect possible complications (Figs. 1, 2).

### Statistical analysis

Histopathological results were evaluated and divided into two diagnostic categories. The first diagnostic category included the diagnosis of a malignant pleural tumour confirmed by lesion regression after undergoing oncological therapy or lesion progression despite received therapy. Patients with the above mentioned findings were included in the group of patients with true-positive (TP) results.

The second diagnostic category included patients with negative histopathological findings or those who have been definitively diagnosed with a benign lesion. All patients included in this category were clinically monitored and controlled using non-invasive radiological methods for 6–12 months. In the observed period, if the lesion size regressed or remained the same, they were included in the group of patients with true-negative (TN) results. Patients with negative histopathological findings, whose clinical course and following CT findings referred to the malignant aetiology of pleural disease, were included in the group of patients with false-negative (FN) results. The definitive diagnosis of patients in the FN category was determined by video-assisted thoracoscopic (VATS) biopsy or open surgical biopsy.

Positive histopathological diagnoses after surgery in the case of an operable pleural tumour, regression of lesion size after undergoing oncological therapy, or progression of findings despite therapy were taken as confirmation of diagnosis.

The diagnostic contribution of this method was determined by tests of overall diagnostic accuracy, sensitivity, specificity, and positive and negative predictive values.

## Results

In this study, no complications were noted among all 56 patients who underwent CT-guided TCNB. The smallest biopsied pleural plaque was 4 mm thick, while the mean thickness of the pleural lesions was 6.2 mm.

The histopathological diagnoses of tissue samples obtained from biopsy procedures are shown in Table 1. The most common malignant diagnosis among patients in this study was malignant pleural mesothelioma, diagnosed in 19 patients (33.9%). Division of mesothelioma by cell type shows that the epithelioid was the most common (13 patients, 23.2%), followed by sarcomatoid (4 patients, 7.1%) and biphasic (2 patients, 3.6%) mesothelioma. The second most frequent diagnosis was metastasis of primary tumours, diagnosed in 14 patients (25%).

**Table 1:** *Histopathological diagnosis for 56 biopsies*

Histopathological diagnosis	N of patients	%
**Primary malignancy**	**19**	**33.9**
Mesothelioma	19	33.9
**Secondary malignancy**	**14**	**25**
Lung adenocarcinoma	8	14.3
Breast adenocarcinoma	2	3.6
Colon adenocarcinoma	1	1.8
Melanoma	1	1.8
Non-Hodgkin lymphoma	1	1.8
Adenoid cystic carcinoma	1	1.8
**Benign lesions**	**17**	**30.4**
Chronic fibrinous pleuritis	6	10.7
Chronic inflammatory infiltrate	5	8.9
Asbestosis	4	7.1
Fibrous pleural plaque	2	3.6
**False-inconclusive findings**	**6**	**10.7**

Among all, the malignant disease was identified in 33 patients (58.9%) included in the group with true-positive results. The final benign diagnosis was confirmed in 17 patients (30.4%), so they were counted as true-negative results. A total of 6 (10.7%) inconclusive findings of negative or non-representative samples were categorised as false-negative results since TCNB did not determine the diagnosis. In these six patients, the CT morphology of the pleural lesions was highly suspected to be a malignant tumour, which was later confirmed by VATS biopsy or open surgery. The final diagnoses of all six false-negative biopsies were epithelioid mesothelioma.

Based on the obtained data, the diagnostic contribution of CT-guided TCNB of pleural lesions in this study was: sensitivity 84.6% (33 of 39), specificity 100% (17 of 17), PPV 100% (33 of 33), and NPV 73.9% (17 of 23), and diagnostic accuracy 89.3% (50 of 56) (Table 2).

**Table 2:** *Measures of diagnostic accuracy*

Diagnostic contribution	N / %
True positive	33 / 58.9
False positive	0 / 0
True negative	17 / 30.4
False negative	6 / 10.7
Sensitivity	84.6
Specificity	100
Positive predictive value	100
Negative predictive value	73.9
Diagnostic accuracy	89.3

## Discussion

This retrospective study included transthoracic core needle biopsies (TCNB) of 56 small pleural lesions with thickness up to 10 mm in patients with loculated pleural effusion, which was considered a protective factor for complications. Major complications of TCNB of pleural lesions include pneumothorax, haemorrhage or needle tract bleeding, and haemothorax. In this study, no complications occurred.

Maskell et al. [14] and Adams et al. [15] also reported a zero-complication rate of CT-guided TCNB of pleural lesions in the presence of pleural effusion. In the study of 33 TCNB, Welch et al. [16] also reported no complications, nor did they mention the presence of pleural effusion.

Benamore et al. [17] reported that 4.7% of CT-guided pleural biopsies were associated with pneumothorax and 7.5% with significant bleeding up the trocar needle. They also mentioned that none of the patients with pneumothorax had pleural effusion.

The complication rates in the Cao et al. study [18] were 6.5% for pneumothorax, 8.7% for haemorrhage, and 1.1% for haemothorax. They also reported that 25% of cases had pleural effusion but did not state whether it had any impact on the rate of complications. They both stated that the observed pneumothoraces may have resulted from the introduction of air by the biopsy or drain rather than direct communication with the airway.

In the Niu et al. study [19], pneumothorax was observed in 16% of patients and chest pain in 2% of patients, while haemothorax was detected in one patient (1%). The same authors reported a lesion size/pleural thickening as a significant risk factor; pleural effusion, present in 40.9% of patients, was noted as a significant protective factor for pneumothorax.

As already mentioned, the mandatory criterion for this study was the presence of loculated pleural effusion, greater than 20 mm, localised immediately adjacent to the pleural lesion. Consequently, the needle did not penetrate the visceral pleural sheet nor enter the lung parenchyma, avoiding the most common complications (pneumothorax and needle tract bleeding).

In the recent publications, to our knowledge, only Adams et al. [20] reported minor haemoptysis (1%) as a complication of CT-guided TCNB of the pleura.

In our study, the sensitivity of CT-guided transthoracic biopsy was 84.6%, specificity 100%, PPV 100%, and NPV 73.9%, while diagnostic accuracy was 89.3%. The overall diagnostic contribution of TCNB in our study is comparable with the results of other reports. In the available literature, all authors [14, 15, 17–21] stated the same specificity and PPV of 100%, while sensitivity [14, 16–19, 21] ranged from 75 to 90.9%, NPV from different studies [14, 15, 17–19, 21] varied from 58.3 to 88.1%, while overall diagnostic accuracy [15, 18, 21] ranged from 89.2 to 94.6%.

In the most recent study, Park et al. [11] reported a similar diagnostic yield of US-guided TCNB of pleural lesions smaller than 20 mm; accuracy, sensitivity, specificity, PPV, and NPV were 85.4%, 84.8%, 100.0%, 100%, and 21.1%, respectively. The only complication in the previously mentioned study was pneumothorax, which was found in 3.9% of patients.

Khosla et al. [22] compared US-guided biopsy with CT-guided biopsy of pleural-based lung lesions. They showed that the US guidance method has an equivalent diagnostic yield, with fewer complications and significantly reduced procedure time. It is important to emphasise that the study included larger lesions, with a mean lesion size of 4.9 and 5.6 cm for CT and US guidance, respectively. Furthermore, Lee et al. [12] concluded that US guidance should be considered for biopsy of peripheral lung and pleural lesions larger than 10 mm.

It should be noted that we obtained our results by analysing lesions smaller than 10 mm and compared them with studies that included differently sized pleural plaques because we did not find studies showing exclusively biopsy results of small pleural lesions in recent publications.

The limitation of our study may be due to a retrospective design with a possible risk of selection bias. Also, all procedures were performed by two radiologists with significant experience in performing CT-guided pleural biopsies.

## Conclusions

The results from our study, which include zero complication rate and significant diagnostic contribution, indicate that CT-guided transthoracic core needle biopsy (TCNB) is a useful method for diagnosing suspected small pleural lesions in the presence of loculated pleural effusion as a protective factor for complications.

## Data Availability

The datasets used and/or analysed during the current study are available from the corresponding author on reasonable request.

## Abbreviations

CT: Computed tomography
TCNB: Transthoracic core needle biopsy
PPV: Positive predictive value
NPV: Negative predictive value
PACS: Picture archiving and communication system
PT: Prothrombin time
INR: International normalised ratio
PTT: Partial thromboplastin time
TP: True positive
FP: False positive
TN: True negative
FN: False negative
VATS: Video–assisted thoracoscopic biopsy
N: Number (of patients)
